# On the importance of monitoring and valuing all forms of biodiversity

**DOI:** 10.1371/journal.pbio.3000039

**Published:** 2018-11-13

**Authors:** Martin A. Schlaepfer

**Affiliations:** Institute of Environmental Sciences, University of Geneva, Geneva, Switzerland

## Abstract

Pauchard et al claim that non-native species should not be granted conservation value, as this could hinder effort to curtail novel introductions. In this response, Schlaepfer counters that the positive contributions of non-native species to biodiversity and conservation must be included to provide a complete and objective snapshot to policy makers.

Which dimensions of biodiversity do we, as a society, value and wish to protect? In my view, biodiversity is worthy of protection for at least three reasons. First, all living organisms have a fundamental (intrinsic) right to existence. Second, from an anthropocentric perspective, biodiversity provides the basis for a good human life through its ecosystem services (now called nature’s contributions to people) [1]. Third, biodiversity provides the raw material for evolution by natural selection that allows populations to adapt to changing environmental conditions. The Convention on Biological Diversity and the Aichi targets capture these three dimensions in recognition of their importance for sustaining a good life on earth for present and future generations.

Strangely, biodiversity indicators used to describe the state of the environment and measure progress toward the Aichi targets consider only native species [2]. Most biodiversity assessments and indicators provide no justification for why non-native species are excluded, so one can only speculate as to the reasons for this oversight. One possibility is that biodiversity indicators were established by biologists who, at the time, implicitly assumed that species had no value outside of their historical range and, further, that this opinion was universal.

Today, most conservation biologists understand the potential risks that non-native species represent, but a growing number also appreciate that non-native species contribute to all three dimensions of biodiversity mentioned above [3–6]. It becomes interesting to consider not only the circumstances under which the effects of non-native species could be net positive but also the possibility that they might even play critical roles in supporting human life in the near future.

After reading Pauchard and colleagues’ piece [7], it is unclear to me where they stand. They appear to support the idea of including non-native species in assessments that describe the state of the environment as long as the origin of species is specified. But they appear to resist the idea of integrating non-native species into conservation indicators, based on the concern that this will dilute the message that non-native species can sometimes cause harm. Implicitly, they are willing to disregard all non-native species based on the assumption that, as a group, they do more harm than good.

As a conservation biologist, I am aware that a minority of non-native species can become problematic, and I favour policies that carefully screen species to prevent the importation of novel species likely to cause undesirable change [8]. But the mere possibility that some non-native species will become problematic does not give us license to ignore the positive contributions of non-native species already established in their host ecosystems [e.g., 3, 4, 9]. Just as one should consider both positive and negative impacts when evaluating non-native species [3,10], conservation policies should avoid simplistic strategies (“non-native = bad”) and instead should discourage only those non-native species with clear net negative effects, and preserve or enhance those that are net positive.

Conservation targets integrate both scientific and normative components. An interdisciplinary approach such as the ecosystem services method, which can incorporate biological, social, health, and economic factors, and the preferences of various stakeholders, could be used to prioritise the components of nature (e.g., species, functions, ecosystems, landscapes) that are important at various scales of governance [2,11,12]. Arguments to exclude non-native species from such a process overlook opportunities for the future, run counter to internationally agreed upon goals (e.g., Aichi targets), and leave the impression that a group of biologists is attempting to impose their personal beliefs (namely that species only have value within their historical range) on the rest of society.
